# Supplementary material for: A Tissue Engineered Model of Aging: Interdependence and Cooperative Effects in Failing Tissues
Source: Sci Rep. 2017 Jul 11;7:5051. doi: 10.1038/s41598-017-05098-2 (PMC5506028; doi:10.1038/s41598-017-05098-2)
Supplement: Supplementary file 1 — Supplementary Information [file 41598_2017_5098_MOESM1_ESM.docx]

**Supplementary Information for**

**A Tissue Engineered Model of Aging: Interdependence and Cooperative Effects in Failing Tissues**

1. Acun^1^, D. C. Vural^2^, P. Zorlutuna^1, 3^*

**Supplementary Figures and Figure Legends**

**
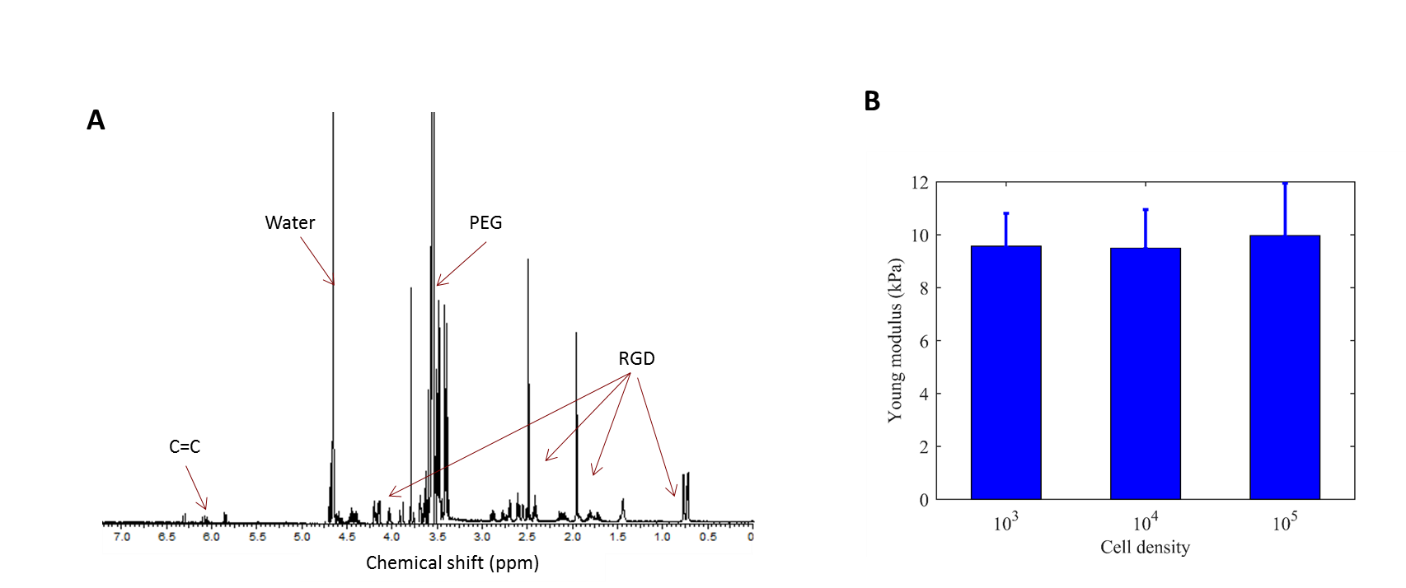
**

**Fig. S1.** (A) The ^1^H-NMR spectrum of PEG-RGD. (B) The young’s modulus (kPa) of PEG-RGD hydrogels with 10^3^, 10^4^, and 10^5^ cells encapsulated per construct.

***
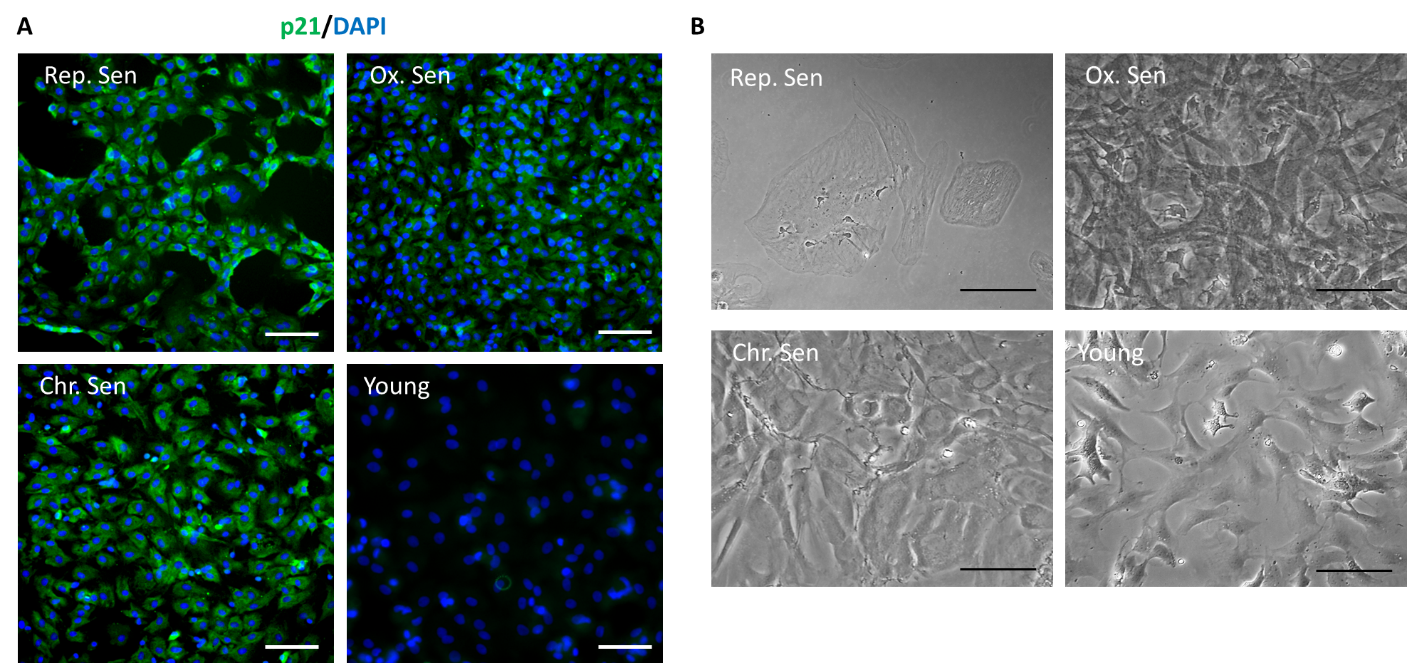
***

**Fig. S2.** (A) The p21 immunostaining of pre-aged and young cells (p21:green, nuclei:blue). (B) Bright field images of pre-aged and young cells. (Scale bar= 100 μm)

**
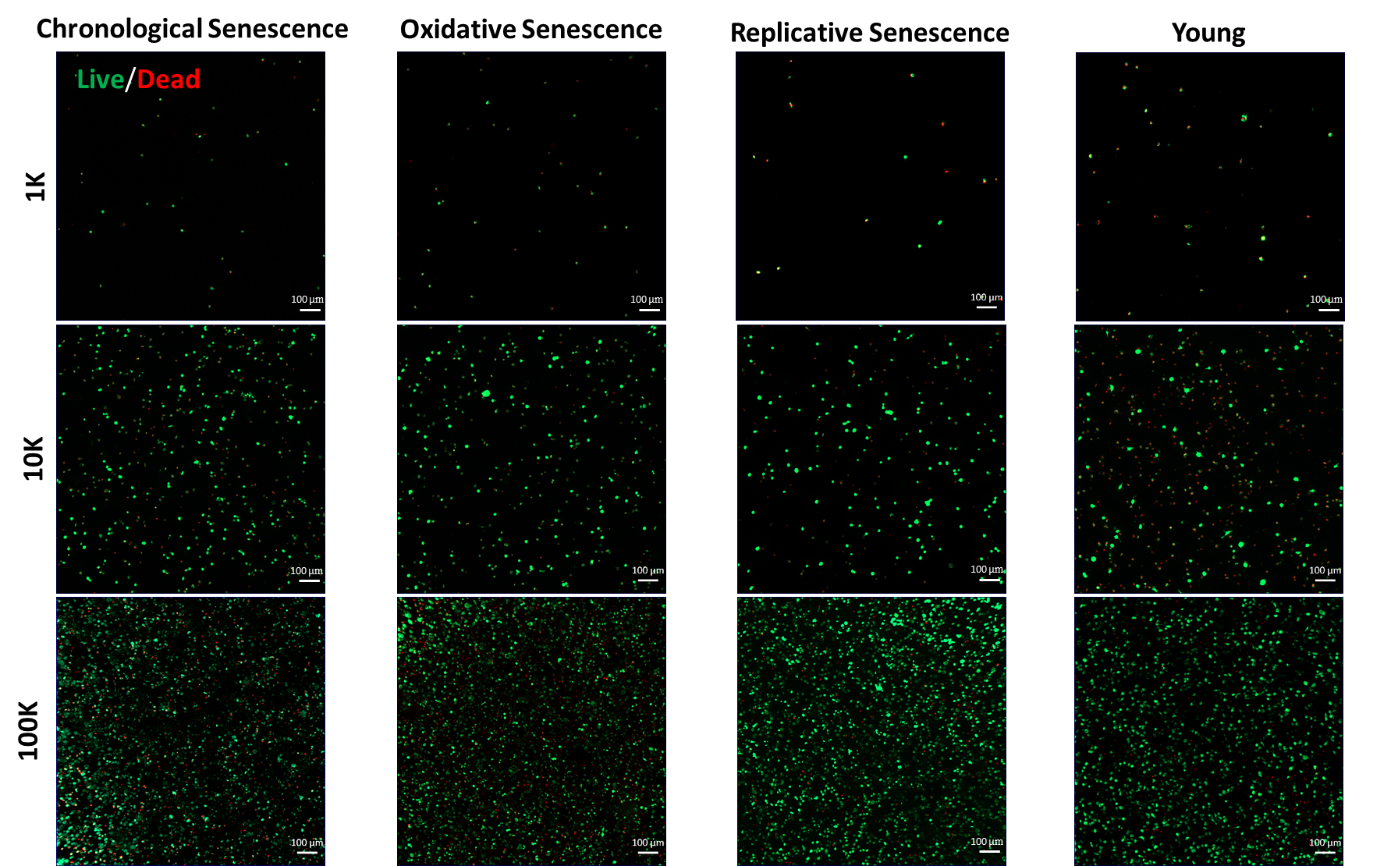
**

**Fig. S3.** The live/dead assay images of pre-aged and young tissue constructs at different cell encapsulation densities (Scale bars=100 μm).


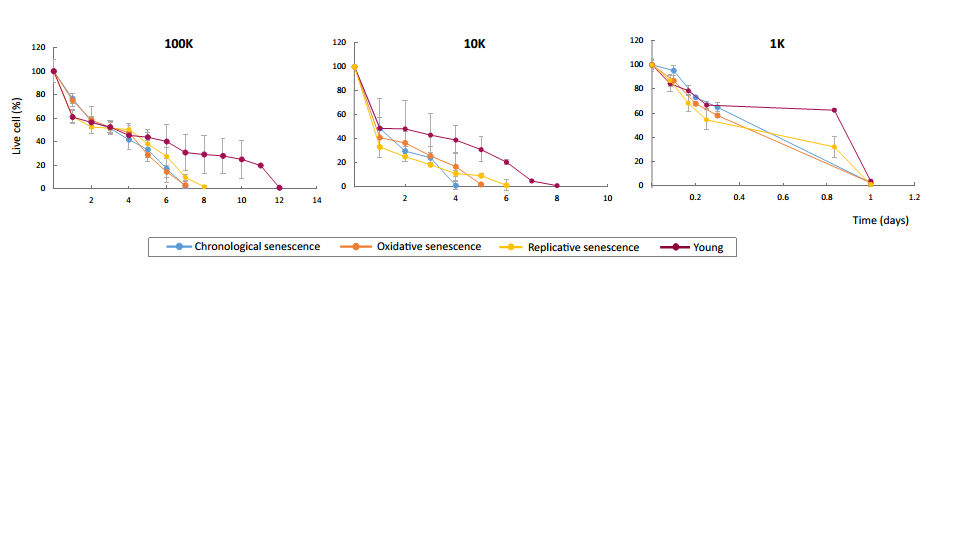


Fig. S4. The change in live cell (%) over time of synthetic tissues made from young or pre-aged cells with different population densities under oxidative stress.


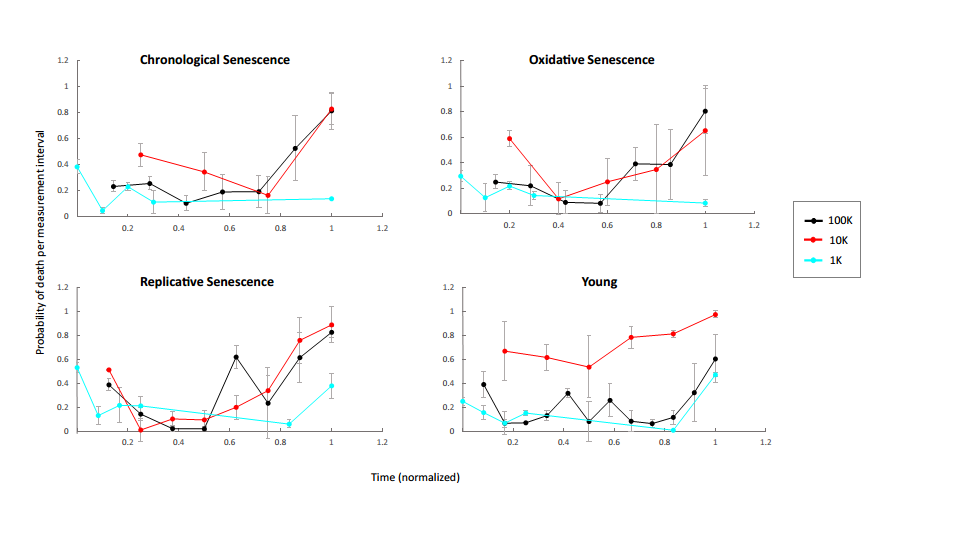


Fig. S5. The change in probability of death of synthetic tissues made from young or pre-aged cells with different population densities under oxidative stress, over time normalized with respect to the measurement intervals.


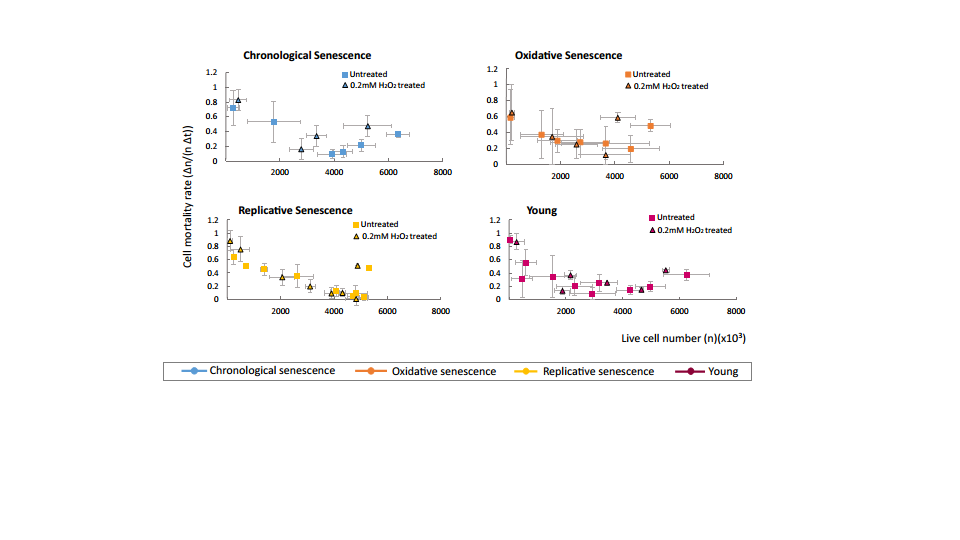


**Fig. S6.** The change in cell mortality rate with respect to live cell number of synthetic tissues made from 10K young or pre-aged cells under oxidative stress.


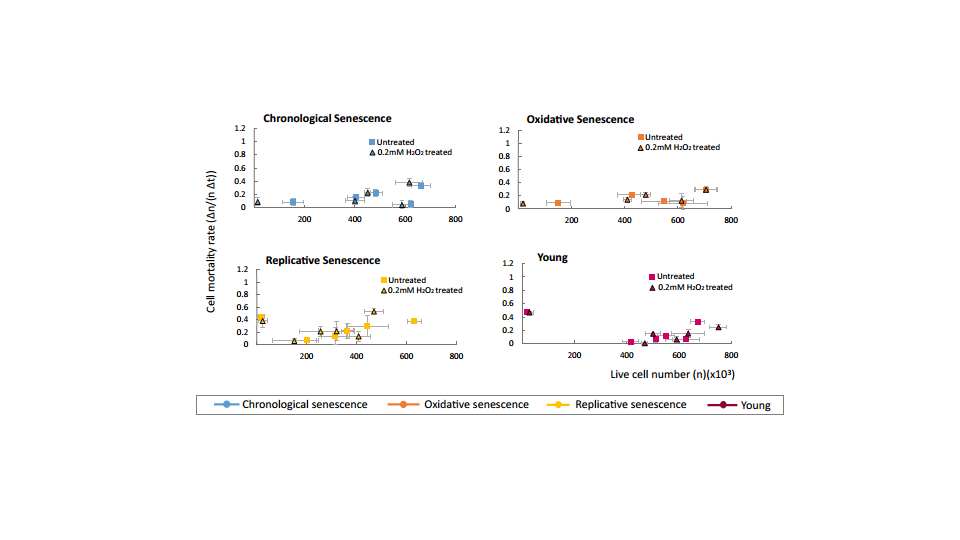


Fig. S7. The change in cell mortality rate with respect to live cell number of synthetic tissues made from 1K young or pre-aged cells under oxidative stress.


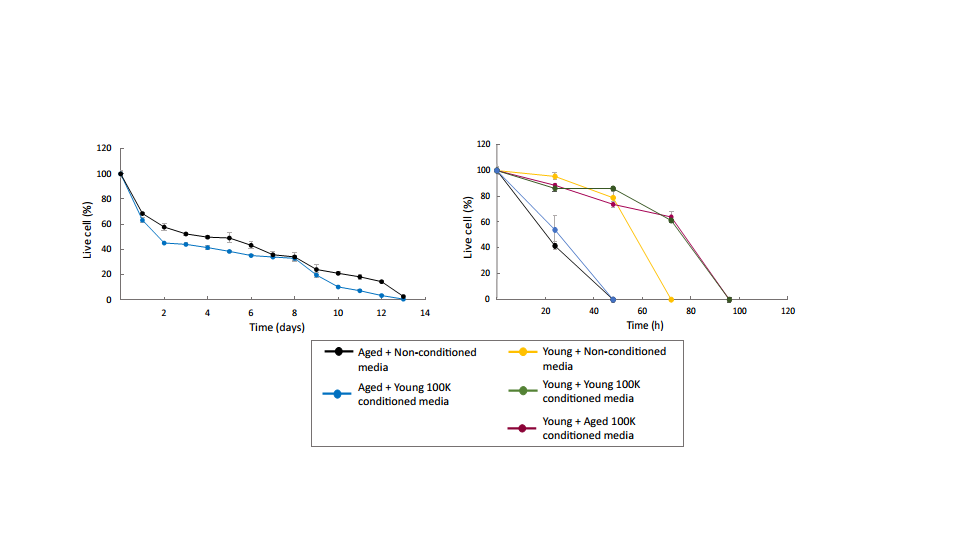


Fig. S8. The change in live cell percent over time of (A) synthetic tissues with 100K pre-aged cells receiving 100K young tissue conditioned or non-conditioned media, and (B) the live cell percentages of synthetic tissues made from 1K young or pre-aged cells receiving 100K young or 100K aged tissue conditioned or non-conditioned media, under oxidative stress.


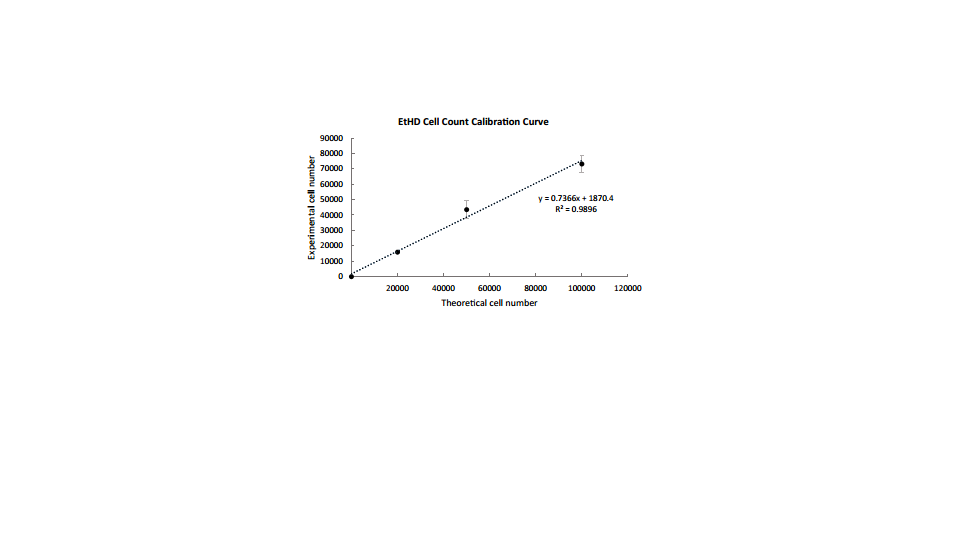


Fig. S9. The calibration curve for cell counting using the images from z-stack slices of EtHD-1 labeled tissues.
